# Clinicopathological correlation of ARID1A status with HDAC6 and its related factors in ovarian clear cell carcinoma

**DOI:** 10.1038/s41598-019-38653-0

**Published:** 2019-02-20

**Authors:** Mitsutake Yano, Tomomi Katoh, Mariko Miyazawa, Masaki Miyazawa, Naoki Ogane, Maiko Miwa, Kosei Hasegawa, Hisashi Narahara, Masanori Yasuda

**Affiliations:** 1 Department of Pathology, Saitama Medical University International Medical Centre, Saitama, Japan; 2 Departments of Obstetrics and Gynaecology, Oita University Faculty of Medicine, Oita, Japan; 3 Department of Obstetrics and Gynaecology, Tokai University School of Medicine, Kanagawa, Japan; 4 Division of Pathology, Ashigarakami Hospital, Kanagawa, Japan; 5 Department of Gynaecologic Oncology, Saitama Medical University International Medical Centre, Saitama, Japan

## Abstract

Ovarian clear cell carcinoma (OCCC) is associated with a frequent loss in ARID1A function. ARID1A reportedly suppresses histone deacetylase (HDAC)6 in OCCC directly. Here, we evaluated the clinical significance of HDAC6 expression and its related factors in terms of ARID1A status. Immunohistochemical expression of HDAC6, hypoxia inducible factors-1α (HIF-1α), programmed death-1 ligand (PD-L1), CD44 (cancer stem cell marker), and ARID1A was analysed for 106 OCCC patients. High nuclear HDAC6 expression correlated with patient death (*p* = 0.038). In the multivariate analysis of overall survival, surgical status (complete or incomplete resection) (hazard ratio (HR) = 17.5; *p* = <0.001), HDAC6 nuclear expression (HR = 1.68; *p* = 0.034), and PD-L1 expression (HR = 1.95; *p* = 0.022) were the independent prognostic factors. HDAC6 upregulation and ARID1A loss did not necessarily occur simultaneously. High HDAC6 expression was associated with poor prognosis in OCCC with ARID1A loss; this was not observed without ARID1A loss. HDAC6 expression showed a significant positive correlation with HIF-1α, PD-L1, and CD44. In OCCC, HDAC6 involvement in prognosis depended on ARID1A status. HDAC6 also led to immuno- and hypoxia- tolerance and cancer stem cell phenotype. HDAC6 is a promising therapeutic target for OCCC with loss of ARID1A.

## Introduction

Ovarian clear cell carcinoma (OCCC) ranks second as the leading cause of death from epithelial ovarian cancer (EOC)^1^ and is associated with the worst prognosis among the major subtypes of EOC when diagnosed at the advanced stages^2,3^. Typically, OCCC exhibits a low response rate to the platinum-based standard chemotherapy used to treat EOC. To date, we have proposed several therapeutic target substances and pathways for OCCC^4,5^. The most common somatic mutation identified in OCCC is that in *ARID1A* (46–57%)^6,7^, a factor that promotes SWI/SNF-mediated chromatin remodelling and displays one of the highest mutation rates among the epigenetic regulators in cancers^8,9^. Therapeutic strategies that harness this genetic characteristic are being explored^10^.

Histone deacetylases (HDACs) are chromatin-modifying enzymes involved in the regulation of many aspects of cell biology, including tissue differentiation, apoptosis, migration, mitosis, and angiogenesis via the deacetylation of histone or non-histone proteins^11^. Eighteen HDAC family members have been identified in humans^11^. The pan-HDAC inhibitor has been demonstrated to exhibit cytotoxic effects in various cancers, including EOC^12^. However, its activities of targeting multiple HDACs lead to various toxicities, which limits its application in the treatment of cancers^13^. More selective and effective HDAC inhibitors are therefore required in cancer therapy. In our previous study, HDAC6 and HDAC7 showed higher expression in OCCC than in other histological subtypes of EOC, and were expected to be poor prognostic factors^14^. Although HDAC7-selective inhibitors are yet to be well-developed, HDAC6-selective inhibitors are clinically used as antitumour agents.

**Figure 1:**
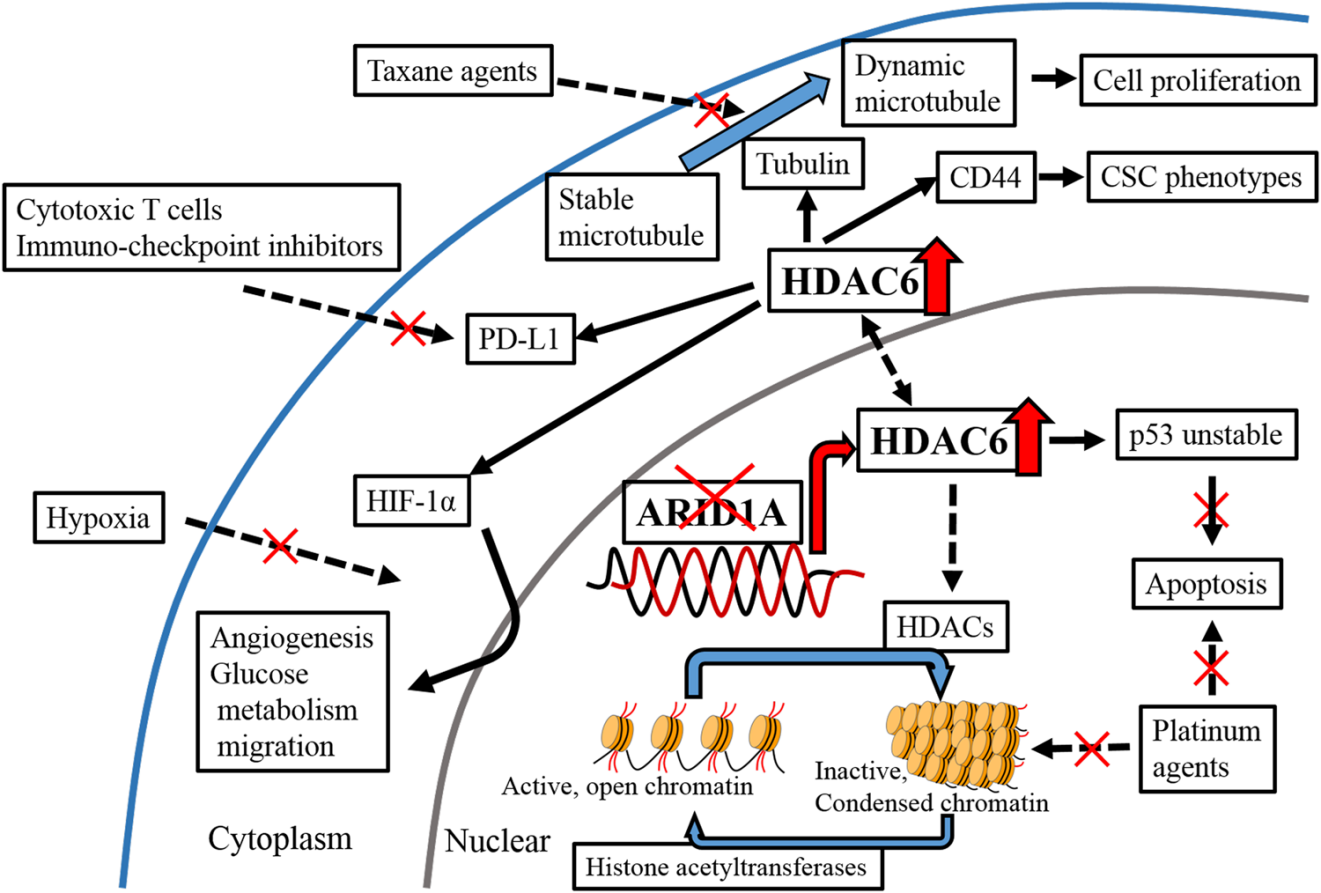
Scheme of HDAC6 functions: When ARID1A loss occurs, suppression of HDAC6 is relieved. In the nucleus, HDAC6 destabilizes p53 by deacetylation, and suppresses apoptosis. As a member of the HDAC family, HDAC6 inactivates chromatin by deacetylation of the core histones. These effects of HDAC6 in the nucleus are responsible for the resistance to platinum agents. In the cytoplasm, HDAC6 leads to cell proliferation, angiogenesis, glucose metabolism, glucose transport, and CSC phenotypes via tubulin, HIF-1α, PD-L1, and CD44. In addition, HDAC6 result in tolerance to taxane agents, cytotoxic T cells, immuno-checkpoints inhibitors, and hypoxic stress.

HDAC6 increases deacetylated α-tubulin levels. This in turn enhances microtubule dynamics and leads to cancer cell growth (Fig. 1)^15,16^. HDAC6 is associated with several chemoresistant factors (Fig. 1) and upregulation of programmed death-1 ligand (PD-L1), which leads to cancer immune tolerance^17^. Hypoxia inducible factor-1α (HIF-1α) protein expression, transcriptional activity^18^, and tumour angiogenesis^19^ are induced by HDAC6, and the cancer stem cell phenotype is maintained by HDAC6 via CD44^20^. HDAC6-selective inhibitors are currently in clinical trials for multiple myeloma^21,22^. Recently, Bitler *et al*.^23^ identified that ARID1A directly suppresses HDAC6 in OCCC and provided evidence that HDAC6 may be a promising therapeutic target in *ARID1A*-mutated cancers. However, the significance of the association between HDAC6 and ARID1A has not been well documented in clinical samples. Herein, we investigated the significance of HDAC6 as a therapeutic target in OCCC and the usefulness of ARID1A as its biomarker using clinical samples.

## Results

### Clinicopathological characteristics and immunohistochemical (IHC) expressions

Table 1 lists the characteristics of the patients. The age of patients ranged from 32 to 80 years, with the average being 55.7 years. All patients included in the study were Japanese. Patients with OCCC were classified after surgery as International Federation of Obstetrics and Gynaecology (FIGO) stage I (n = 71), stage II (n = 16), stage III (n = 17), and stage IV (n = 2). A total of 90 (84.9%) patients underwent complete surgical resection, while 85 (80.2%) patients underwent adjuvant chemotherapy after surgery. Lymphadenectomy was performed in 62 patients (58.5%), 9.7% of which had lymph nodes metastasis. All adjuvant chemotherapies administered to patients contained platinum-agents, 84.7% of which were combined with taxane-agents. The median follow-up duration was 54.2 months for survivors (range, 10–121 months). Recurrence and death were observed in 32 (30.2%) and 23 (21.7%) patients, respectively.

**Table 1:** Clinicopathological characteristics of OCCC patients (n = 106).

Variable	N (%)
**Age**	
Median (range)	55.7 (32–80)
>56	50 (47.2)
≤56	56 (52.8)
**FIGO stage**	
I	71 (67.0)
II	16 (15.1)
III	17 (16.0)
IV	2 (1.9)
**Surgical procedures**	
TAH + BSO + OM	93 (87.8)
BSO + OM	1 (0.9)
USO + OM	11 (10.4)
OM	1 (0.9)
**Lymphadenectomy**	
Yes	62 (58.5)
No	44 (41.5)
**Surgical status**	
Complete resection	90 (84.9)
Incomplete resection	16 (15.1)
**Adjuvant chemotherapy**	
Yes	85 (80.2)
Paclitaxel + Carboplatin	60
Docetaxel + Carboplatin	12
Irinotecan + Cisplatin	12
Gemcitabine + Carboplatin	1
No	21 (19.8)
**Recurrence**	
Yes	32 (30.2)
No	74 (69.8)
**Death**	
Yes	23 (21.7)
No	83 (78.3)

FIGO, the International Federation of Obstetrics and Gynaecology; TAH, total abdominal hysterectomy; BSO, bilateral salpingo-oophorectomy; OM, omentectomy; USO, unilateral salpingo-oophorectomy.

A positive expression of ARID1A was observed in 54 patients (50.9%), while a high expression of HDAC6 was observed in 59 (55.7%) and 20 (18.9%) (nucleus and cytoplasm, respectively) patients; high expression was observed in 60 (56.6%), 7 (6.6%), and 24 (22.6%), patients for HIF-1α, PD-L1, and CD44, respectively. The correlations between patient characteristics and IHC expressions are shown in Table 2. No correlation was found between all IHC expressions (HDAC6, HIF-1α, PD-L1, ARID1A, and CD44) and age, FIGO stage, and surgical status. A high expression of CD44 was correlated with recurrence (*p* = 0.018), while a high expression of HDAC6 (nucleus), HIF-1α, PD-L1, and CD44 correlated with death (*p* = 0.038, 0.047, 0.038, and 0.036, respectively). There was no significant correlation between ARID1A loss and any of the available clinicopathological parameters.

**Table 2:** The correlations between patient characteristics and IHC expressions.

		Age (mean = 55.7)			FIGO stage			Residual tumour			Recurrence			Death		
		<56	≥56	*p* value	I + II	III + IV	*p* value	Yes	No	*p* value	Yes	No	*p* value	Yes	No	*p* value
ALL		50	56		87	19		90	16		32	74		23	83	
ARID1A	Positive	27	27	0.345	43	11	0.340	44	10	0.233	15	39	0.367	10	44	0.283
	Loss	23	29		44	8		46	6		17	35		13	39	
HDAC6N	High	26	33	0.301	49	10	0.482	49	10	0.376	20	39	0.237	**17**	**42**	**0**.**038**
	Low	24	23		38	9		41	6		12	35		**6**	**41**	
HDAC6C	High	8	12	0.322	16	4	0.504	17	3	0.647	8	12	0.212	6	14	0.237
	Low	42	44		71	15		73	13		24	62		17	69	
HIF-1α	High	29	31	0.469	49	11	0.554	50	10	0.408	22	38	0.073	**17**	**43**	**0**.**047**
	Low	21	25		38	8		40	6		10	36		**6**	**40**	
PD-L1	High	3	4	0.564	5	2	0.367	5	2	0.285	4	3	0.121	**4**	**3**	**0**.**038**
	Low	47	52		82	17		85	14		28	71		**19**	**80**	
CD44	High	12	12	0.466	18	6	0.229	18	6	0.114	**12**	**12**	**0**.**018**	**9**	**15**	**0**.**036**
	Low	38	44		69	13		72	10		**20**	**62**		**14**	**68**	

HDAC6N, histone deacetylase 6 nuclear expression; HDAC6C, HDAC6 cytoplasmic expression; HIF-1α, hypoxia inducible factor-1α; PD-L1, programmed death-1 ligand. *p* value < 0.05 is shown in bold.

### Correlation with survival and IHC expressions

In the univariate analysis using the Cox proportional hazards model, high expression of PD-L1 and CD44, FIGO stage, and surgical status were found as the prognostic factors for progression free survival (PFS) and overall survival (OS) (Table 3). In the multivariate analysis, high expression of HIF-1α (hazard ratio (HR) = 1.75; 95% CI, 1.17 to 2.61, *p* = 0.006), PD-L1 (HR = 2.34; 95% CI, 1.28 to 4.30, *p* = 0.006), and CD44 (HR = 1.62; 95% CI, 1.10 to 2.38, *p* = 0.014), and surgical status (complete vs. incomplete resection) were demonstrated as the independent prognostic factors for PFS. High expression of HDAC6 (nuclear) (HR = 1.68; 95% CI, 1.04 to 2.70, *p* = 0.034) and PD-L1 (HR = 1.95; 95% CI, 1.10 to 3.45, *p* = 0.022), and surgical status were the independent prognostic factors for OS (Table 3).

**Table 3:** Univariable and multivariable analyses using the Cox proportional hazards model of overall survival for OCCC patients.

Variable	Univariate analysis			Multivariate analysis		
	HR	95% CI	*p* value	HR	CI	*p* value
Age	1.01	0.45–2.30	0.978			
ARID1A	1.51	0.66–3.45	0.329			
HDAC6N	1.56	0.98–2.48	0.063	**1**.**68**	**1**.**04–2**.**70**	**0**.**034**
HDAC6C	1.30	0.81–2.07	0.273			
HIF-1α	1.56	0.98–2.49	0.061			
PD-L1	**2**.**08**	**1**.**21–3**.**58**	**0**.**009**	**1**.**95**	**1**.**10–3**.**45**	**0**.**022**
CD44	**1**.**63**	**1**.**07–2**.**49**	**0**.**022**			
FIGO stage	**6**.**68**	**2**.**94–15**.**2**	**<0**.**001**			
Residual tumour	**15**.**2**	**6**.**49–35–7**	**<0**.**001**	**17**.**2**	**6**.**90–43**.**5**	**<0**.**001**

HR, hazard ratio; CI, confidence interval; HDAC6N, histone deacetylase 6 nuclear expression; HDAC6C, HDAC6 cytoplasmic expression; HIF-1α, hypoxia inducible factor-1α; PD-L1, programmed death-1 ligand. p value < 0.05 is shown in bold.

### Dependency of HDAC6 and HIF-1α expression on ARID1A

**Figure 2:**
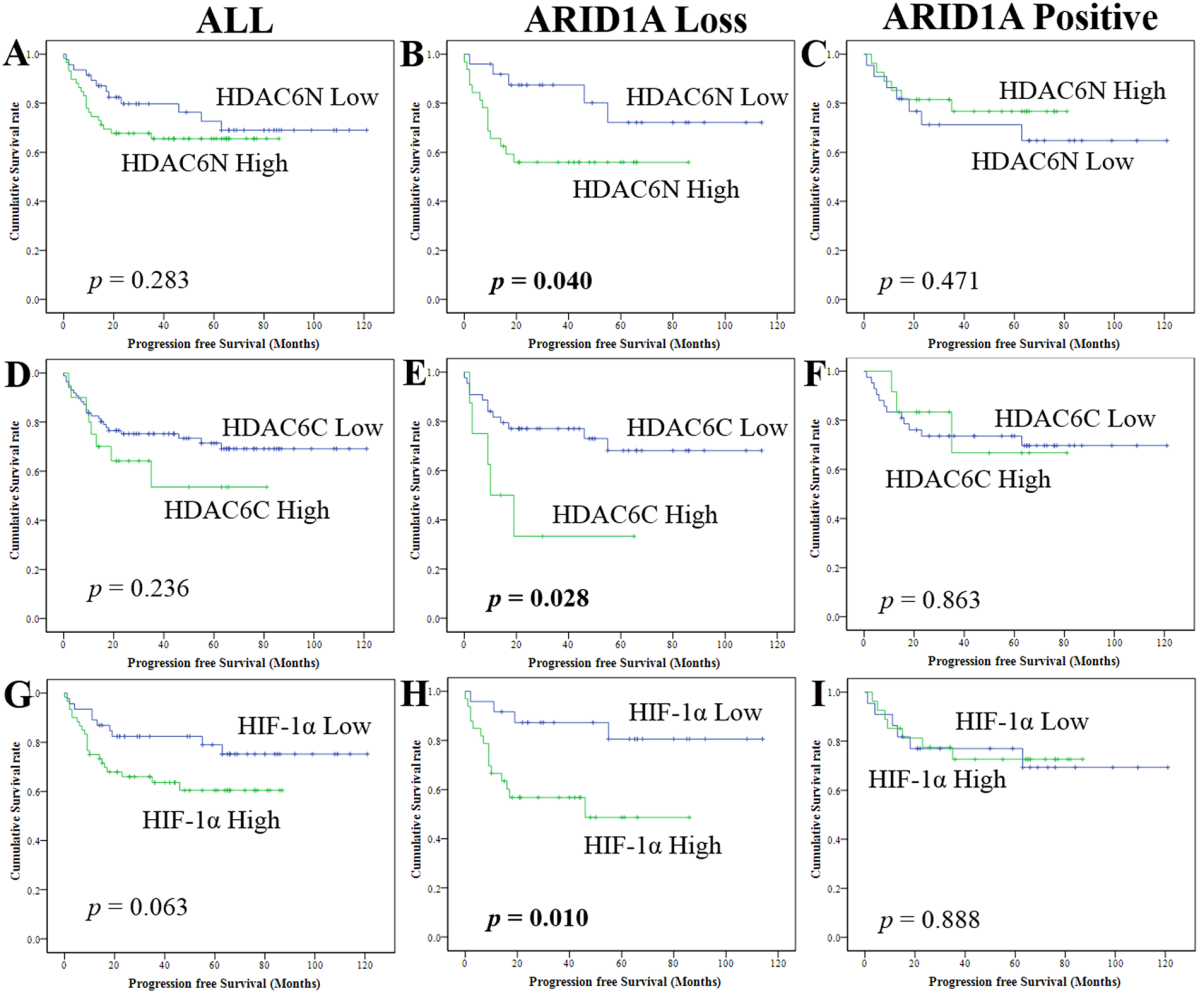
Kaplan-Meier survival analysis: Nuclear HDAC6 in (**A**) all cases, (**B**) with, and (**C**) without ARID1A loss. Cytoplasmic HDAC6 in (**D**) all cases, (**E**) with, and (**F**) without ARID1A loss. HIF-1α in (**G**) all cases, (**H**) with, and (**I**) without ARID1A loss. *p* values, log rank test.

A subgroup analysis was performed in the presence or absence of ARID1A loss (Fig. 2). A high expression of HDAC6 (nucleus, *p* = 0.040; cytoplasm, *p* = 0.028) had an adverse effect on the PFS in patients with ARID1A loss; however, a high expression of HDAC6 had no adverse effect on the PFS in all patients (nucleus, *p* = 0.283; cytoplasm, *p* = 0.236) and in those without ARID1A loss (nucleus, *p* = 0.417; cytoplasm, *p* = 0.863) (Fig. 2A–F). A high expression of HIF-1α (*p* = 0.010) had an adverse effect on the PFS of patients with ARID1A loss; however, this was not observed in all patients (*p* = 0.063) and in patients without ARID1A loss (*p* = 0.888) (Fig. 2G–I).

### Correlation among IHC expressions

**Figure 3:**
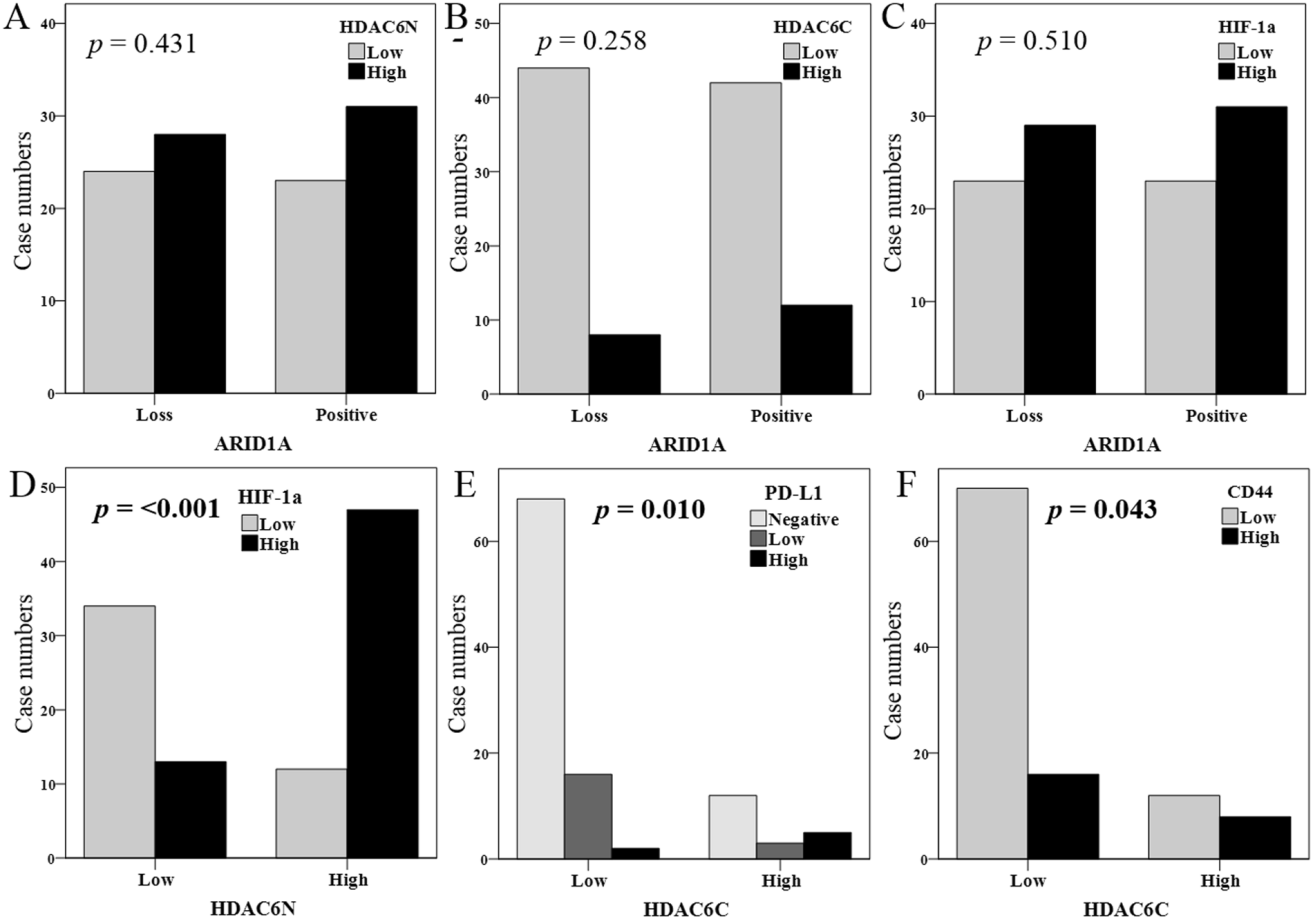
Correlations among IHC expressions, using the Chi-square test.

ARID1A loss also showed a significantly positive correlation with the high expression of PD-L1 (Table 4); however, this was not observed with the high expression of HDAC6 (nucleus, *p* = 0.431, Fig. 3A; cytoplasm, *p* = 0.258, Fig. 3B) and HIF-1α (*p* = 0.510, Fig. 3C). The nuclear high expression of HDAC6 also showed a significantly positive correlation with HIF-1α (*p* = <0.001, Fig. 3D). The cytoplasmic high expression of HDAC6 showed a significantly positive correlation with PD-L1 (*p* = 0.010, Fig. 3E) and CD44 (*p* = 0.043, Fig. 3F).

**Table 4:** Spearman’s correlations among IHC expressions.

		HDAC6N	HDAC6C	HIF-1α	PD-L1	ARID1A	CD44
HDAC6N	Correlation coefficient	1	**0**.**236**	**0**.**521**	0.008	0.036	−0.107
	*p* value		**0**.**015**	**<0**.**001**	0.936	0.715	0.275
HDAC6C	Correlation coefficient		1	−0.064	**0**.**357**	0.087	**0**.**200**
	*p* value			0.513	**<0**.**001**	0.373	**0**.**040**
HIF-1α	Correlation coefficient			1	−0.074	0.017	−0.118
	*p* value				0.452	0.866	0.23
PD-L1	Correlation coefficient				1	**−0**.**271**	**0**.**219**
	*p* value					**0**.**005**	**0**.**024**
ARID1A	Correlation coefficient					1	−0.004
	*p* value						0.965
CD44	Correlation coefficient						1
	*p* value						

HDAC6N, histone deacetylase 6 nuclear expression; HDAC6C, HDAC6 cytoplasmic expression; HIF-1α, hypoxia inducible factor-1α; PD-L1, programmed death-1 ligand. *p* value < 0.05 is shown in bold.

## Discussion

In the present study, OCCC patients with high nuclear expression of HDAC6 had a poor prognosis regardless of FIGO stage and surgical status, the latter of which is a well-known important prognostic factor in EOC. These results suggest that HDAC6 is one of the refractory factors to the standard treatments in OCCC. The standard chemotherapy for EOC is a combination of platinum and taxane agents; however, OCCC patients are resistant to this combination. The deacetylation of alpha-tubulin, induced by HDAC6, decreases the effect of taxane agents as a microtubule-stabilizing agent^24^. When HDAC6 is inhibited, taxane resistance is reversed in EOC cell lines^24,25^. HDAC6 upregulation leads to tumour cisplatin resistance, and depletion of HDAC6 enhances cisplatin-induced DNA damage and apoptosis^26^. HDAC6-selective inhibitors exhibit an anti-tumour effect in breast cancer^27,28^, gastric cancer^19^, multiple myeloma^21,22^, and lymphoma^29^. Therefore, we suggest that HDAC6 is a potentially key therapeutic target for OCCC. Notably, HDAC6-selective inhibitors are well-tolerated and show minimal toxicity in clinical trials^21,22^. HDAC6-selective inhibitors may therefore improve the efficacy and adverse effects such as kidney failure^30^ and peripheral neuropathy^31^ that often accompany the standard chemotherapy for EOC.

The present study also showed the coexistence of an upregulation in HDAC6 and ARID1A loss, leading to a shorter survival for OCCC patients than for patients having either one of the two factors; these activities do not necessarily happen simultaneously. Bitler *et al*.^23^ showed that an HDAC6-selective inhibitor (ACY-1215, ricolinostat) suppressed the proliferation of *ARID1A*-mutated OCCC cell lines and improved the survival of mice bearing *ARID1A*-mutated OCCC compared to that of mice bearing *ARID1A*-wild type OCCC. Fukumoto *et al*.^32^ also reported that the pan-HDAC inhibitor improved the survival of mice bearing *ARID1A*-mutated OCCC, while Gupta *et al*.^33^ demonstrated that HDAC inhibitors responded to *ARID1A*-mutated urothelial cancers when compared to that in *ARID1A*-wild type urothelial cancers, in two clinical trials. These observations therefore indicate that ARID1A status is an important biomarker in the treatment of HDAC. A high PD-L1 expression demonstrated a positive correlation with high HDAC6 cytoplasmic expression and ARID1A loss. When HDAC6 is inhibited, immunotherapy response is enhanced with PD-L1 blockage^34–36^. Shen *et al*.^37^ showed that treatment with an anti-PD-L1 antibody reduced tumour burden and prolonged survival of *ARID1A*-mutated mice; this was not observed in the *ARID1A*-wild type EOCs. HIF-1α and CD44 also showed a positive correlation with HDAC6. Therefore, HDAC6 may serve as a therapeutic target for OCCC with ARID1A loss associated with PD-L1, HIF-1α, and CD44 expression. Given that a loss in ARID1A is frequent in cancers^9^, the present findings may have implications beyond the established OCCC.

Our study had several limitations. The sample size used in this study was small, and the survival analysis was only performed with a few events. However, when considering the low incidence of OCCC, the present study included a relatively large number of patients. Before drawing a conclusion based on the results of this study, further confirmation is warranted via a multi-ethnic population study and on a larger scale. Secondly, the present study consisted solely of semi-quantitated IHC analysis and lacked both quantitative protein analysis and molecular correlations; the molecular correlations in OCCC between ARID1A and HDAC6 have already been reported^23^. The novelty of the present study is its verification of the findings in clinical samples. However, further studies are required to quantitatively analyse HDAC6 protein and mRNA expression.

In conclusion, the involvement of HDAC6 in OCCC prognosis was demonstrated to depend on the ARID1A status. HDAC6 was observed to function as a promising therapeutic target for OCCC with ARID1A loss, in close association with immuno-modulation, response to hypoxia, and cancer stem cell phenotype. HDAC6-selective inhibitors are expected to have safe and synergistic effects when combined with the current standard chemotherapy for EOC.

## Methods

### Patients and samples

Patient electronic medical charts from the Saitama Medical University International Medical Centre from 2007 to 2016 were reviewed under the approval of the institutional review board (IRB number, 16–257). All methods were performed in accordance with the 1975 Declaration of Helsinki. All tumour specimens in the pathological analysis were obtained with informed consent (or a formal waiver of consent) with an approval from the Ethics Committee of our hospital. We recruited 106 patients with OCCC, whose tumours were surgically resected and pathologically confirmed. The clinicopathological characteristics of these cases, such as age, recurrence/PFS, death/OS, FIGO stage, surgical status (complete resection or incomplete resection), and treatment methods were reviewed.

### IHC staining

IHC expression of HDAC6, ARID1A, HIF-1α, PD-L1, and CD44 was analysed using tissue microarray (KIN-2, AZUMAYA, Tokyo, Japan). Tissue microarray was generated from 2 cylindrical cores that were 3.0 mm in diameter in each block; these were punched out of paraffin-embedded tissue blocks corresponding to the representative histological findings, and then inserted into a recipient block. A total of 106 tissue blocks were cut into 4-μm serial sections, and were each run through an automated system by Dako Autostainer Link 48 (Agilent technologies, CA, USA) as per the manufacturer’s protocol. The following primary antibodies were used: polyclonal rabbit anti-HDAC6 (dilution, 1:500; ab1440, Abcam, Cambridge, UK), polyclonal rabbit anti-HIF-1α (dilution, 1:100; NB100–479; Novus Biologicals, CO, USA), monoclonal rabbit anti-PD-L1 (dilution, 1:100; 28-8 pharmDx; Dako North America, CA, USA), monoclonal rabbit anti-ARID1A (dilution, 1:1000; ERP13501-73; Abcam, Cambridge, UK), and monoclonal mouse anti-CD44 (dilution, 1:200; 156-3C11; Thermo Fisher Scientific, MA, USA). For all antibodies, the Target Retrieval Solution (pH 9.0, HDAC6 and CD44; pH 6.0, ARID1A, HIF-1α, and PD-L1) was applied for antigen retrieval at 98 °C for 20 min. Sections were incubated with the primary antibodies at 25 °C for 60 min, followed by incubation with a secondary antibody (EnVision FLEX/HRP, Agilent technologies, CA, USA) at 25 °C for 30 min. The chromogen reaction was performed with diaminobenzidine plus the H_2_O_2_ substrate at 25 °C for 10 min.

### Interpretation of IHC results

**Figure 4:**
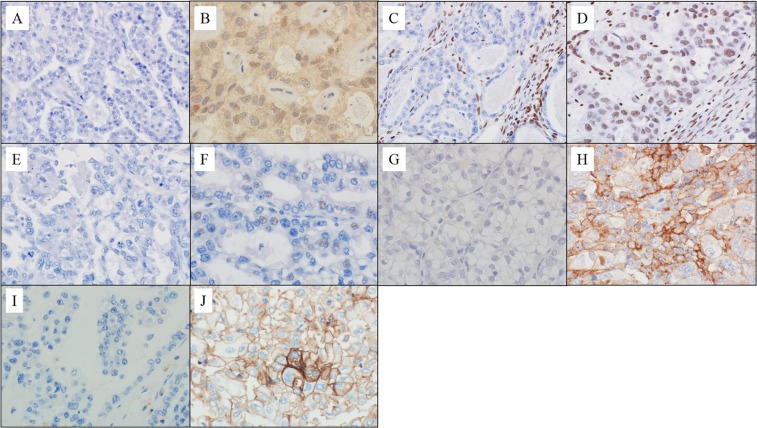
IHC expressions. HDAC6 ((**A**) low; (**B**) high), ARID1A ((**C**) loss; (**D**) positive), HIF-1α ((**E**) low; (**F**) high), PD-L1 ((**G**) low; (**H**) high), and CD44 ((**I**) low; (**J**) high).

IHC evaluation was performed by one pathologist (Masanori Yasuda) and one physician (Mitsutake Yano) with subspecialties in gynaecological oncology; both of them were blinded to the clinicopathological parameters (Fig. 4). The following four-tiered scoring scheme was used: negative (0%), weak (1–50%), moderate (51–80%), and marked (81–100%). To optimize the PFS and OS differences, the raw data were binarised for statistical analysis as follows: in HDAC6, HIF-1α, PD-L1, and CD44. The moderate and marked expression were grouped as high-level, whereas the completely negative and weak expression were grouped as low level. For ARID1A, the completely negative expression was categorised as the loss-group, whereas the weak, moderate, and marked expressions were categorised as positive groups. This categorisation was based on the evidence that the complete absence of ARID1A expression is significantly correlated with its mutation status^38^.

### Statistical analysis

IHC expressions and the clinicopathological parameters were assessed using the Pearson chi-square test or the Fisher exact test. Spearman’s rank correlation was used to determine whether there was a positive or a negative correlation between the factors. Univariable survival analysis was performed by generating Kaplan-Meier curves, and differences between the groups were assessed using the log rank statistic. Univariable and multivariable survival analyses were performed using the Cox proportional hazards model. All analyses were performed using SPSS v24.0 (SPSS Inc, IL, USA). *P* values < 0.05 were considered significant.
